# *N*-(2-Bromo-4-methyl­phen­yl)-2-(5-methyl-2-phenyl­pyrazolo­[1,5-*a*]pyrimidin-7-yl)acetamide

**DOI:** 10.1107/S1600536813011811

**Published:** 2013-05-04

**Authors:** Ibtissam Bassoude, Sabine Berteina-Raboin, El Mokhtar Essassi, Gérald Guillaumet, Lahcen El Ammari

**Affiliations:** a Laboratoire de Chimie Organique Hétérocyclique URAC21, Pôle de Compétences Pharmacochimie, Université Mohammed V-Agdal, Avenue Ibn Battouta, BP 1014, Rabat, Morocco; b Institut de Chimie Organique et Analytique, Université d’Orléans, UMR CNRS 6005, BP 6759, 45067 Orléans Cedex 2, France; c Institute of Nanmaterials and Nanotechnology, MASCIR, Rabat, Morocco; d Laboratoire de Chimie du Solide Appliquée, Université Mohammed V-Agdal, Faculté des Sciences, Avenue Ibn Battouta, BP 1014, Rabat, Morocco

## Abstract

The fused pyrazole and pyrimidine rings in the title compound, C_22_H_19_BrN_4_O, are almost coplanar, their planes being inclined to one another by 2.08 (13)°. The dihedral angles formed by the mean plane of the fused ring system and the phenyl and benzene rings are 16.21 (4) and 82.84 (4)°, respectively. An intra­molecular N—H⋯N hydrogen bond is observed. In the crystal, mol­ecules form inversion dimers *via* pairs of C—H⋯O hydrogen bonds. π–π inter­actions, with centroid–centroid distances of 3.4916 (9) Å, connect the dimers into a three-dimensional network.

## Related literature

For pharmacological and biochemical properties of pyrazolo­[1,5-*a*]pyrimidine derivatives, see: Selleri *et al.* (2005 ▶); Almansa *et al.* (2001 ▶); Suzuki *et al.* (2001 ▶); Chen *et al.* (2004 ▶). For related structures, see: Bassoude *et al.* (2013*a* ▶,*b* ▶).
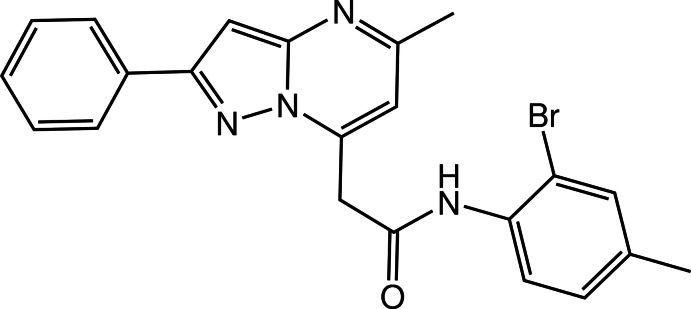


## Experimental

### 

#### Crystal data

C_22_H_19_BrN_4_O
*M* *_r_* = 435.32
Monoclinic, 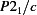

*a* = 9.8102 (6) Å
*b* = 7.2915 (4) Å
*c* = 27.0162 (14) Å
β = 92.942 (3)°
*V* = 1929.95 (19) Å^3^
*Z* = 4
Mo *K*α radiation
μ = 2.15 mm^−1^
*T* = 296 K
0.42 × 0.33 × 0.25 mm

#### Data collection

Bruker X8 APEXII area-detector diffractometer
Absorption correction: multi-scan (*SADABS*; Bruker, 2009 ▶) *T*_min_ = 0.739, *T*_max_ = 0.867
29996 measured reflections
6371 independent reflections
3830 reflections with *I* > 2σ(*I*)
*R*_int_ = 0.044

#### Refinement

*R*[*F* ^2^ > 2σ(*F* ^2^)] = 0.041
*wR*(*F* ^2^) = 0.107
*S* = 1.02
6371 reflections
253 parameters
H-atom parameters constrained
Δρ_max_ = 0.41 e Å^−3^
Δρ_min_ = −0.62 e Å^−3^

### 

Data collection: *APEX2* (Bruker, 2009 ▶); cell refinement: *SAINT* (Bruker, 2009 ▶); data reduction: *SAINT*; program(s) used to solve structure: *SHELXS97* (Sheldrick, 2008 ▶); program(s) used to refine structure: *SHELXL97* (Sheldrick, 2008 ▶); molecular graphics: *ORTEP-3 for Windows* (Farrugia, 2012 ▶); software used to prepare material for publication: *PLATON* (Spek, 2009 ▶) and *publCIF* (Westrip, 2010 ▶).

## Supplementary Material

Crystal structure: contains datablock(s) I, global. DOI: 10.1107/S1600536813011811/rz5060sup1.cif

Structure factors: contains datablock(s) I. DOI: 10.1107/S1600536813011811/rz5060Isup2.hkl

Supplementary material file. DOI: 10.1107/S1600536813011811/rz5060Isup3.cml

Additional supplementary materials:  crystallographic information; 3D view; checkCIF report

## Figures and Tables

**Table 1:** Hydrogen-bond geometry (Å, °)

*D*—H⋯*A*	*D*—H	H⋯*A*	*D*⋯*A*	*D*—H⋯*A*
N4—H4*A*⋯N2	0.86	2.17	2.927 (2)	147
C14—H14⋯O1^i^	0.93	2.43	3.189 (2)	139

Symmetry code: (i) 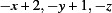.

**Fig. 1.**
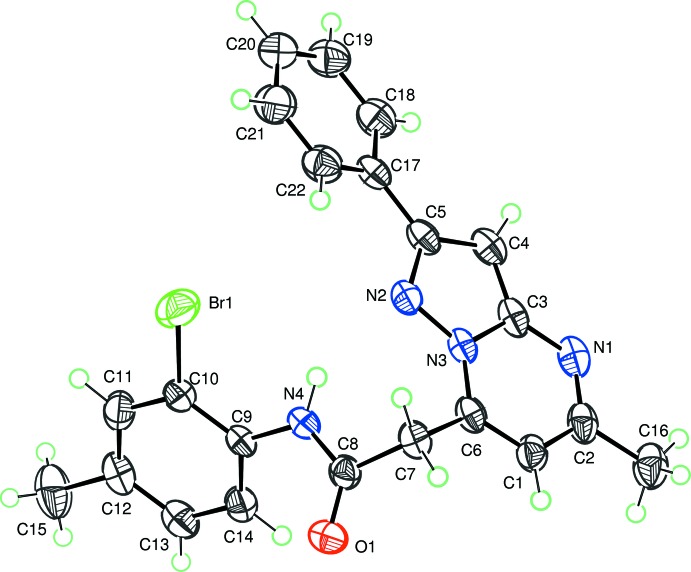
The molecular structure of the title compound with displacement ellipsoids drawn at the 50% probability level. H atoms are represented as small circles of arbitrary radii.

**Fig. 2.**
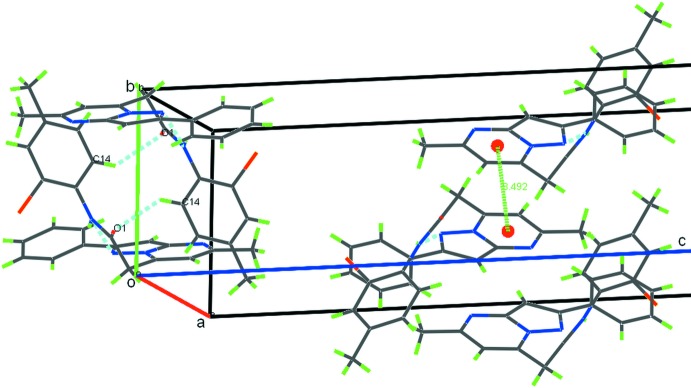
Partial crystal packing of the title compound showing C14–H14A···O1 and N4–H4···N2 hydrogen bonds as blue dashed lines and a π···π contact as green line. The red spheres represent the centroids of the pyrimidine rings.

## supplementary crystallographic information

### Comment

Pyrazolo[1,5-*a*]pyrimidines have attracted considerable interest because of their biological activity. For instance, they are known for their potent utility as selective peripheral benzodiazepine receptor ligands (Selleri *et al.*, 2005), COX-2 selective inhibitors (Almansa *et al.*, 2001), HMG-CoA reductase inhibitors (Suzuki *et al.*, 2001) and CRF1 antagonists (Chen *et al.*, 2004). Our research group targeted at the development of heterocycles with a bridgehead nitrogen atom such the title compound and related compounds recently published (Bassoude *et al.* 2013*a*, 2013*b*).

The crystal structure of the title compound is built up from two fused five (N2/N3/C3–C5) and six-membered (N1/N3/C1–C3/C6) rings linked to one phenyl ring (C17–C22) and to a 2-bromo-4-methylphenyl ring (Br1/C9–C14) through an acetamide group as shown in Fig. 1. The pyrazole and pyrimidine rings are essentially planar with the maximum deviation of 0.0039 (15) and 0.0076 (15) Å for atom C3. The plane through the fused ring system makes a dihedral angles of 16.21 (4)° and 82.84 (4)° with the phenyl ring and with the 2-bromo-4-methylphenyl ring, respectively.

In the crystal (Fig. 2) molecules form inversion dimers *via* pairs of C14—H14···O1 hydrogen bonds. In addition, π–π interactions connect the dimers into a three-dimensional network, with centroid–centroid distances of 3.4916 (9) Å. An intramolecular N4—H4A···N2 hydrogen bond my help to define the conformation of the molecule.

### Experimental

To a solution of 2-bromo-4-methylaniline (0.1 ml, 0.78 mmol) in 4 ml dichloromethane, under argon and at 273 K, were added 0.8 ml of trimethylaluminium in toluene (2M, 1.6 mmol), then the mixture was stirred for 15 min followed by addition of 0.2 g (0.68 mmol) of 7-ethoxycarbonyl-methyl-5-methylpyrazolo[1,5-*a*]pyrimidine. The reaction mixture was stirred to room temperature for 30 min then heated to reflux for 5 h. After evaporation of solvent under reduced pressure, the residue was extracted with CH_2_Cl_2_ and washed with a saturated NaCl solution. The combined organic layers were dried with MgSO_4_ and concentrated under vacuum. The residue was purified on silica gel by column chromatography using mixture of petroleum ether and ethyl acetate (9:1 *v*/*v*) as eluent. The compound was recrystallized from a mixture of cyclohexane/ diethyl ether (1:1 *v*/*v*) to give colourless crystals.

### Refinement

All H atoms could be located in a difference Fourier map and were treated as riding, with C—H = 0.93–0.97 Å, N–H = 0.86 Å, and with *U*_iso_(H) = 1.2 *U*_eq_(C, N) or 1.5 *U*_eq_(C) for methyl H atoms. Two outliers (1 0 0; 0 0 2) were omitted from the last refinement cycles

### Crystal data

**Table:**

C_22_H_19_BrN_4_O	*F*(000) = 888
*M**_r_* = 435.32	*D*_x_ = 1.498 Mg m^−^^3^
Monoclinic, *P*2_1_/*c*	Mo *K*α radiation, λ = 0.71073 Å
Hall symbol: -P 2ybc	Cell parameters from 6371 reflections
*a* = 9.8102 (6) Å	θ = 2.5–31.4°
*b* = 7.2915 (4) Å	µ = 2.15 mm^−^^1^
*c* = 27.0162 (14) Å	*T* = 296 K
β = 92.942 (3)°	Block, colourless
*V* = 1929.95 (19) Å^3^	0.42 × 0.33 × 0.25 mm
*Z* = 4	

### Data collection

**Table:**

Bruker X8 APEXII area-detector diffractometer	6371 independent reflections
Radiation source: fine-focus sealed tube	3830 reflections with *I* > 2σ(*I*)
Graphite monochromator	*R*_int_ = 0.044
φ and ω scans	θ_max_ = 31.4°, θ_min_ = 2.5°
Absorption correction: multi-scan (*SADABS*; Bruker, 2009)	*h* = −14→14
*T*_min_ = 0.739, *T*_max_ = 0.867	*k* = −10→10
29996 measured reflections	*l* = −39→39

### Refinement

**Table:**

Refinement on *F*^2^	Primary atom site location: structure-invariant direct methods
Least-squares matrix: full	Secondary atom site location: difference Fourier map
*R*[*F*^2^ > 2σ(*F*^2^)] = 0.041	Hydrogen site location: difference Fourier map
*wR*(*F*^2^) = 0.107	H-atom parameters constrained
*S* = 1.02	*w* = 1/[σ^2^(*F*_o_^2^) + (0.0467*P*)^2^ + 0.2795*P*] where *P* = (*F*_o_^2^ + 2*F*_c_^2^)/3
6371 reflections	(Δ/σ)_max_ = 0.001
253 parameters	Δρ_max_ = 0.41 e Å^−^^3^
0 restraints	Δρ_min_ = −0.62 e Å^−^^3^

### Special details

**Table:**

Geometry. All e.s.d.'s (except the e.s.d. in the dihedral angle between two l.s. planes) are estimated using the full covariance matrix. The cell e.s.d.'s are taken into account individually in the estimation of e.s.d.'s in distances, angles and torsion angles; correlations between e.s.d.'s in cell parameters are only used when they are defined by crystal symmetry. An approximate (isotropic) treatment of cell e.s.d.'s is used for estimating e.s.d.'s involving l.s. planes.
Refinement. Refinement of *F*^2^ against all reflections. The weighted *R*-factor *wR* and goodness of fit *S* are based on *F*^2^, conventional *R*-factors *R* are based on *F*, with *F* set to zero for negative *F*^2^. The threshold expression of *F*^2^ > σ(*F*^2^) is used only for calculating *R*-factors(gt) *etc*. and is not relevant to the choice of reflections for refinement. *R*-factors based on *F*^2^ are statistically about twice as large as those based on *F*, and *R*- factors based on all data will be even larger.

### Fractional atomic coordinates and isotropic or equivalent isotropic displacement parameters (Å^2^)

**Table:**

	*x*	*y*	*z*	*U*_iso_*/*U*_eq_	
C1	0.6173 (2)	0.8274 (2)	−0.03898 (7)	0.0377 (4)	
H1	0.6836	0.8688	−0.0597	0.045*	
C2	0.4907 (2)	0.7594 (2)	−0.05997 (7)	0.0389 (4)	
C3	0.41574 (19)	0.7044 (2)	0.01713 (7)	0.0363 (4)	
C4	0.3365 (2)	0.6608 (2)	0.05592 (7)	0.0389 (4)	
H4	0.2478	0.6155	0.0539	0.047*	
C5	0.41646 (19)	0.6987 (2)	0.09908 (7)	0.0345 (4)	
C6	0.64217 (19)	0.8324 (2)	0.01096 (7)	0.0329 (4)	
C7	0.76984 (19)	0.9055 (2)	0.03730 (7)	0.0366 (4)	
H7A	0.7446	0.9745	0.0661	0.044*	
H7B	0.8141	0.9894	0.0154	0.044*	
C8	0.87117 (19)	0.7571 (2)	0.05392 (7)	0.0330 (4)	
C9	0.89437 (18)	0.4831 (2)	0.10641 (7)	0.0332 (4)	
C10	0.89659 (19)	0.4341 (2)	0.15591 (7)	0.0361 (4)	
C11	0.9568 (2)	0.2718 (3)	0.17291 (8)	0.0422 (5)	
H11	0.9557	0.2416	0.2063	0.051*	
C12	1.0184 (2)	0.1551 (3)	0.14043 (8)	0.0436 (5)	
C13	1.0160 (2)	0.2037 (3)	0.09087 (8)	0.0432 (5)	
H13	1.0567	0.1265	0.0685	0.052*	
C14	0.9547 (2)	0.3641 (3)	0.07366 (8)	0.0397 (4)	
H14	0.9538	0.3925	0.0401	0.048*	
C15	1.0869 (3)	−0.0203 (3)	0.15829 (10)	0.0691 (7)	
H15A	1.1706	0.0085	0.1766	0.104*	
H15B	1.1060	−0.0955	0.1303	0.104*	
H15C	1.0274	−0.0854	0.1793	0.104*	
C16	0.4645 (2)	0.7598 (3)	−0.11515 (8)	0.0512 (5)	
H16C	0.4995	0.8709	−0.1287	0.077*	
H16A	0.3680	0.7523	−0.1229	0.077*	
H16B	0.5091	0.6563	−0.1292	0.077*	
C17	0.38277 (19)	0.6745 (2)	0.15115 (7)	0.0361 (4)	
C18	0.2742 (2)	0.5647 (3)	0.16391 (9)	0.0470 (5)	
H18	0.2209	0.5064	0.1392	0.056*	
C19	0.2447 (2)	0.5415 (3)	0.21290 (9)	0.0541 (6)	
H19	0.1721	0.4670	0.2209	0.065*	
C20	0.3217 (2)	0.6275 (3)	0.25011 (9)	0.0533 (6)	
H20	0.3013	0.6116	0.2831	0.064*	
C21	0.4294 (2)	0.7376 (3)	0.23797 (9)	0.0518 (5)	
H21	0.4818	0.7966	0.2628	0.062*	
C22	0.4595 (2)	0.7604 (3)	0.18897 (8)	0.0452 (5)	
H22	0.5326	0.8345	0.1812	0.054*	
N1	0.39309 (17)	0.6994 (2)	−0.03282 (6)	0.0405 (4)	
N2	0.54163 (15)	0.7640 (2)	0.08894 (6)	0.0338 (3)	
N3	0.53930 (15)	0.76798 (18)	0.03862 (6)	0.0317 (3)	
N4	0.82608 (16)	0.6428 (2)	0.08896 (6)	0.0370 (4)	
H4A	0.7499	0.6695	0.1016	0.044*	
O1	0.98349 (14)	0.74725 (19)	0.03713 (5)	0.0453 (3)	
Br1	0.81300 (3)	0.58958 (3)	0.201758 (8)	0.05961 (10)	

### Atomic displacement parameters (Å^2^)

**Table:**

	*U*^11^	*U*^22^	*U*^33^	*U*^12^	*U*^13^	*U*^23^
C1	0.0391 (11)	0.0356 (9)	0.0378 (10)	0.0071 (8)	−0.0030 (9)	0.0039 (8)
C2	0.0459 (12)	0.0288 (8)	0.0409 (11)	0.0120 (8)	−0.0089 (9)	−0.0027 (7)
C3	0.0315 (10)	0.0304 (8)	0.0458 (12)	0.0060 (7)	−0.0103 (9)	−0.0036 (7)
C4	0.0268 (10)	0.0370 (9)	0.0523 (12)	0.0007 (8)	−0.0044 (9)	−0.0031 (8)
C5	0.0282 (10)	0.0278 (8)	0.0473 (11)	0.0048 (7)	−0.0008 (8)	−0.0028 (7)
C6	0.0303 (10)	0.0275 (8)	0.0405 (10)	0.0056 (7)	−0.0035 (8)	0.0011 (7)
C7	0.0330 (10)	0.0341 (9)	0.0420 (11)	0.0014 (8)	−0.0036 (8)	0.0021 (7)
C8	0.0294 (10)	0.0381 (9)	0.0309 (9)	0.0008 (7)	−0.0032 (8)	−0.0041 (7)
C9	0.0223 (9)	0.0400 (9)	0.0371 (10)	0.0042 (7)	−0.0009 (8)	0.0022 (7)
C10	0.0291 (10)	0.0442 (10)	0.0350 (10)	0.0004 (8)	0.0005 (8)	0.0002 (8)
C11	0.0409 (12)	0.0462 (10)	0.0387 (11)	−0.0011 (9)	−0.0055 (9)	0.0086 (8)
C12	0.0366 (11)	0.0381 (9)	0.0549 (13)	0.0032 (8)	−0.0102 (10)	0.0024 (9)
C13	0.0359 (11)	0.0409 (10)	0.0522 (13)	0.0050 (8)	−0.0026 (9)	−0.0077 (9)
C14	0.0345 (11)	0.0466 (10)	0.0377 (11)	0.0048 (8)	−0.0027 (9)	−0.0010 (8)
C15	0.0758 (19)	0.0495 (12)	0.0796 (18)	0.0205 (13)	−0.0183 (15)	0.0084 (12)
C16	0.0619 (15)	0.0482 (11)	0.0422 (12)	0.0110 (10)	−0.0118 (11)	−0.0065 (9)
C17	0.0277 (10)	0.0335 (9)	0.0470 (12)	0.0077 (7)	0.0010 (8)	−0.0020 (8)
C18	0.0373 (12)	0.0483 (11)	0.0551 (13)	−0.0024 (9)	0.0001 (10)	−0.0032 (9)
C19	0.0418 (13)	0.0624 (13)	0.0588 (15)	−0.0058 (11)	0.0085 (11)	0.0066 (11)
C20	0.0467 (14)	0.0681 (14)	0.0455 (13)	0.0084 (11)	0.0074 (11)	0.0030 (10)
C21	0.0453 (13)	0.0632 (13)	0.0466 (13)	−0.0024 (11)	0.0013 (10)	−0.0085 (10)
C22	0.0366 (11)	0.0490 (11)	0.0501 (13)	−0.0034 (9)	0.0015 (10)	−0.0048 (9)
N1	0.0393 (10)	0.0358 (8)	0.0451 (10)	0.0055 (7)	−0.0107 (8)	−0.0041 (7)
N2	0.0292 (8)	0.0355 (7)	0.0362 (9)	0.0033 (6)	−0.0027 (7)	−0.0015 (6)
N3	0.0276 (8)	0.0296 (7)	0.0371 (9)	0.0048 (6)	−0.0056 (7)	−0.0012 (6)
N4	0.0279 (9)	0.0454 (8)	0.0379 (9)	0.0098 (7)	0.0046 (7)	0.0059 (7)
O1	0.0352 (8)	0.0541 (8)	0.0474 (8)	0.0065 (6)	0.0094 (7)	0.0049 (6)
Br1	0.0728 (2)	0.06541 (16)	0.04192 (14)	0.01753 (12)	0.01561 (12)	0.00091 (10)

### Geometric parameters (Å, º)

**Table:**

C1—C6	1.359 (3)	C11—H11	0.9300
C1—C2	1.428 (3)	C12—C13	1.384 (3)
C1—H1	0.9300	C12—C15	1.512 (3)
C2—N1	1.311 (3)	C13—C14	1.385 (3)
C2—C16	1.500 (3)	C13—H13	0.9300
C3—N1	1.357 (2)	C14—H14	0.9300
C3—C4	1.374 (3)	C15—H15A	0.9600
C3—N3	1.396 (2)	C15—H15B	0.9600
C4—C5	1.399 (3)	C15—H15C	0.9600
C4—H4	0.9300	C16—H16C	0.9600
C5—N2	1.358 (2)	C16—H16A	0.9600
C5—C17	1.472 (3)	C16—H16B	0.9600
C6—N3	1.369 (2)	C17—C22	1.387 (3)
C6—C7	1.506 (3)	C17—C18	1.390 (3)
C7—C8	1.521 (2)	C18—C19	1.380 (3)
C7—H7A	0.9700	C18—H18	0.9300
C7—H7B	0.9700	C19—C20	1.377 (3)
C8—O1	1.215 (2)	C19—H19	0.9300
C8—N4	1.353 (2)	C20—C21	1.380 (3)
C9—C10	1.383 (3)	C20—H20	0.9300
C9—C14	1.393 (3)	C21—C22	1.381 (3)
C9—N4	1.412 (2)	C21—H21	0.9300
C10—C11	1.390 (3)	C22—H22	0.9300
C10—Br1	1.8962 (19)	N2—N3	1.359 (2)
C11—C12	1.384 (3)	N4—H4A	0.8600
			
C6—C1—C2	120.76 (19)	C13—C14—C9	120.30 (19)
C6—C1—H1	119.6	C13—C14—H14	119.8
C2—C1—H1	119.6	C9—C14—H14	119.8
N1—C2—C1	122.65 (18)	C12—C15—H15A	109.5
N1—C2—C16	117.63 (19)	C12—C15—H15B	109.5
C1—C2—C16	119.7 (2)	H15A—C15—H15B	109.5
N1—C3—C4	133.01 (18)	C12—C15—H15C	109.5
N1—C3—N3	121.12 (18)	H15A—C15—H15C	109.5
C4—C3—N3	105.85 (16)	H15B—C15—H15C	109.5
C3—C4—C5	105.95 (17)	C2—C16—H16C	109.5
C3—C4—H4	127.0	C2—C16—H16A	109.5
C5—C4—H4	127.0	H16C—C16—H16A	109.5
N2—C5—C4	112.04 (17)	C2—C16—H16B	109.5
N2—C5—C17	118.99 (17)	H16C—C16—H16B	109.5
C4—C5—C17	128.97 (18)	H16A—C16—H16B	109.5
C1—C6—N3	115.66 (17)	C22—C17—C18	118.12 (19)
C1—C6—C7	125.52 (18)	C22—C17—C5	120.65 (18)
N3—C6—C7	118.81 (16)	C18—C17—C5	121.24 (18)
C6—C7—C8	113.78 (14)	C19—C18—C17	120.7 (2)
C6—C7—H7A	108.8	C19—C18—H18	119.7
C8—C7—H7A	108.8	C17—C18—H18	119.7
C6—C7—H7B	108.8	C20—C19—C18	120.7 (2)
C8—C7—H7B	108.8	C20—C19—H19	119.7
H7A—C7—H7B	107.7	C18—C19—H19	119.7
O1—C8—N4	124.06 (17)	C19—C20—C21	119.3 (2)
O1—C8—C7	121.57 (17)	C19—C20—H20	120.4
N4—C8—C7	114.36 (16)	C21—C20—H20	120.4
C10—C9—C14	117.85 (17)	C20—C21—C22	120.1 (2)
C10—C9—N4	121.28 (16)	C20—C21—H21	119.9
C14—C9—N4	120.76 (17)	C22—C21—H21	119.9
C9—C10—C11	121.64 (18)	C21—C22—C17	121.1 (2)
C9—C10—Br1	119.42 (14)	C21—C22—H22	119.4
C11—C10—Br1	118.93 (15)	C17—C22—H22	119.4
C12—C11—C10	120.42 (19)	C2—N1—C3	117.42 (17)
C12—C11—H11	119.8	C5—N2—N3	103.92 (14)
C10—C11—H11	119.8	N2—N3—C6	125.34 (15)
C11—C12—C13	118.01 (18)	N2—N3—C3	112.24 (15)
C11—C12—C15	121.3 (2)	C6—N3—C3	122.38 (16)
C13—C12—C15	120.7 (2)	C8—N4—C9	125.11 (15)
C12—C13—C14	121.77 (19)	C8—N4—H4A	117.4
C12—C13—H13	119.1	C9—N4—H4A	117.4
C14—C13—H13	119.1		

### Hydrogen-bond geometry (Å, º)

**Table:**

*D*—H···*A*	*D*—H	H···*A*	*D*···*A*	*D*—H···*A*
N4—H4*A*···N2	0.86	2.17	2.927 (2)	147
C14—H14···O1^i^	0.93	2.43	3.189 (2)	139

Symmetry code: (i) −*x*+2, −*y*+1, −*z*.
